# Traits and biogeography are key to non‐native plant abundance, but in surprising ways

**DOI:** 10.1111/nph.70483

**Published:** 2025-08-20

**Authors:** Rachael V. Gallagher

**Affiliations:** ^1^ Hawkesbury Institute for the Environment Western Sydney University Locked Bag 1797 Penrith NSW 2751 Australia

**Keywords:** alien species, functional traits, invasive species, leaf economic spectrum, non‐native plant species, plant traits, species abundance

## Abstract

This article is a Commentary on Blumenthal *et al*. (2025), **248**: 1192–1204.

Many thousands of plant species have been shipped, driven, flown (or have accidentally hitched a ride) around the world, resulting in a wide array of non‐native species being present throughout the world. Determining which of these will remain transient elements of the landscape and which will go on to cause expansive environmental, economic, and social impacts is a key goal in invasion biology. In their insightful article published in this issue of *New Phytologist*, Blumenthal *et al*. (2025; pp. 1192–1204) bring together an impressive collection of previously disparate datasets on plant occurrence across the United States to ask whether species traits, biogeographic origin, or the interface between these factors best explain patterns of abundance in non‐native plant species. Answering this question is not just a scientific endeavour: to make the control of invasive species feasible and effective, we need to understand the fundamental mechanisms by which they become problematic (Catford *et al*., 2009). In this way, invasion biology enjoys a particularly tight coupling to real‐world, applied outcomes where science can directly inform management programmes.

*Strikingly consistent differences in the traits of native and non‐native species were found in all three traits examined: specific leaf area (SLA), leaf dry matter content (LDMC), leaf N concentration (Leaf N)*.

Blumenthal *et al*. address elegantly simple questions: does simply being non‐native underpin a species' abundance in plant communities, or are some species set up for success by possessing traits associated with rapidly acquiring and cycling nutrients? Or is it a combination of both origin and traits which shape abundance? To test these ideas, the authors compile and harmonise data on native and non‐native species abundance from almost 70 000 vegetation plots and species inventories across five ecoregions of the continental United States. This alone constitutes a major achievement and is pivotal to the strength of their findings that non‐native species have distinct patterns of abundance, which are not explained by their traits. Data on patterns of plant abundance that span a wide range of different environmental contexts – from forests in the northern and eastern regions of the United States, to the central plains and southern deserts – are essential for confident predictions of overarching patterns. The global literature on invasion biology repeatedly points to the importance of site‐specific context in explaining why some species thrive and become problematic (Catford *et al*., 2022). Using large datasets to understand abundance allows patterns to rise above the idiosyncrasies inherent to particular locations and their ecological context when searching for generalities. While certainly not the first study to make use of large datasets in invasion biology (e.g. see Seabloom *et al*., 2015; Pyšek *et al*., 2017), Blumenthal *et al*.'s work adds depth to our understanding of how traits and origin interact to shape abundance.

Strikingly consistent differences in the traits of native and non‐native species were found in all three traits examined: specific leaf area (SLA), leaf dry matter content (LDMC) and leaf N concentration (Leaf N). These traits index different aspects of plant strategy variation along the leaf economic spectrum (LES). The LES is a means for mapping how plants vary their leaf investment along a single central axis that runs from acquisitive strategies at one extreme (faster leaf turnover and lower construction costs) to conservative strategies at the other (expensive to construct, in terms of key nutrients, but long‐lasting leaves; Wright *et al*., 2004). Blumenthal *et al*. show that non‐native species in the United States are consistently more acquisitive in their strategy by a wide margin (i.e. SLA and Leaf N were higher, and LDMC lower) relative to native species in 77–86% of the plots studied. Importantly, however, non‐native species with these ‘fast’ traits are more abundant where the community they inhabit is also characterised as acquisitive. These findings imply that to limit the impact of non‐native species, they should be deprived of disturbance and the subsequent chances to colonise, lending empirical support to management practices that are already widely used and that limit soil disturbance, such as low‐tilling rates, mulching, or manual removal (where feasible at the early stages of the invasion continuum; Poland *et al*., 2021).

We have come a long way over the past three decades in corralling the resources needed to address ecological questions on such impressive scales. Studies like this one, and others, which span countries, continents or the world, are now routine (e.g. Díaz *et al*., 2016; Bruelheide *et al*., 2018). The insights which come from large synthesis studies are a testament to the investment of time and effort of ecologists and plant scientists the world over – in both the research sector and government agencies – in collecting, sharing and curating data on species traits and occurrence. Many millions of plant trait observations are now available across a range of sources (e.g. TRY, AusTraits, China Trait Database; Kattge *et al*., 2020; Falster *et al*., 2021; Wang *et al*., 2022) and some of the tools used for their curation and harmonisation are being freely shared and reused to facilitate new databasing efforts. Functional trait approaches to understanding the natural world have spread across ecology, showing that taxonomy is not the only means to understand and classify species. However, taxonomy remains the more ‘complete’ approach across the plant kingdom. That is, while we have estimates of key traits for many species, we are short of a comprehensive global plant trait dataset, with notable gaps in the Global South and high densities of observations in North America and Europe (Maitner *et al*., 2023). Whether one has a ‘completist’ view of trait collection will depend on the scientific question at hand. Blumenthal *et al*. had data available from the TRY database for only 19%, 26%, and 16% of species in their study for Leaf N, SLA, and LDMC, respectively. But as these observations were for common species, they could derive plot‐based trait values across 69 441 plots for ≥ 80% of total cover. This is more than enough data to understand overarching patterns in abundance related to traits. However, it is important not to discount the need to understand the ecology of rare species, which may possess trait constellations which can inform us about diversity, invasion dynamics, or extinction risk, and help set conservation and restoration priorities that avoid homogenisation of the global flora. There are good reasons to ‘chase the tail’ of the distribution of key plant traits to inform rare species management. We must also seek to understand intraspecific variation in traits, which may be particularly important in the context of invasion biology (Siefert *et al*., 2015). Traits may shift in species as they jump geographic barriers and move to new environments, and traits may also vary along environmental gradients in response to changes in rainfall, seasonality, and temperature. Trait shifts may affect the relative abundance of species, as noted by Blumenthal *et al*. (see Westerband *et al*., 2021), and the contribution of this variation is not often considered in macroecological studies. Trait variation may also affect the efficacy of management, particularly where traits vary widely and might affect the performance of control measures, such as biocontrol that targets seed size and production.

Finally, the plot‐based datasets on abundance and occurrence of plant species assembled by Blumenthal *et al*. offer a strong foundation for future research in invasion biology. The authors have, for instance, left a door invitingly ajar to the inclusion of other species‐level observations on the nature of introduction effort. Propagule pressure (i.e. the *number* of individuals that are introduced to found a population) is a remarkably consistent predictor of invasion success across regions and kingdoms (Cassey *et al*., 2018), which could partially explain the consistent pattern of higher non‐native plant abundance relative to native species in the Great Plains. This US ecoregion supports a high density of agricultural land and as such is likely to have been subject to repeated introductions of non‐native species as fodder and crops, as well as any accidental introductions of non‐native species associated with these activities. Furthermore, the authors note that several of the most prevalent non‐native plant species in their study have previously been deliberately improved through selective breeding to increase their economic benefits to people. The extent to which this kind of genetic founding effect may shape the competitiveness of non‐native species, and their transition towards invasion, is yet to be synthesised at the scales relevant to creating broad generalisations. Blumenthal's dataset is well‐curated and awaits new additions to dig deeper into these questions.

## Disclaimer

The New Phytologist Foundation remains neutral with regard to jurisdictional claims in maps and in any institutional affiliations.

## References

[nph70483-bib-0001] Blumenthal DM , Diez J , Pearse I , Sofaer HR , Sorte CJB , Barnett D , Beaury EM , Bradley BA , Corbin JD , Dukes JS *et al*. 2025. Why are non‐native plants successful? Consistently fast economic traits and novel origin jointly explain abundance across US ecoregions. New Phytologist 248: 1192–1204. 10.1111/nph.70307 40552507

[nph70483-bib-0002] Bruelheide H , Dengler J , Purschke O , Lenoir J , Jiménez‐Alfaro B , Hennekens S , Botta‐Dukát Z , Chytrý M , Field R , Jansen F *et al*. 2018. Global trait–environment relationships of plant communities. Nature Ecology & Evolution 2: 1906–1917. 30455437 10.1038/s41559-018-0699-8

[nph70483-bib-0003] Cassey P , Delean S , Lockwood JL , Sadowski JS , Blackburn TM . 2018. Dissecting the null model for biological invasions: a meta‐analysis of the propagule pressure effect. PLoS Biology 16: e2005987. 29684017 10.1371/journal.pbio.2005987 PMC5933808

[nph70483-bib-0004] Catford JA , Jansson R , Nilsson C . 2009. Reducing redundancy in invasion ecology by integrating hypotheses into a single theoretical framework. Diversity and Distributions 15: 22–40.

[nph70483-bib-0005] Catford JA , Wilson JR , Pyšek P , Hulme PE , Duncan RP . 2022. Addressing context dependence in ecology. Trends in Ecology & Evolution 37: 158–170. 34756764 10.1016/j.tree.2021.09.007

[nph70483-bib-0006] Díaz S , Kattge J , Cornelissen JH , Wright IJ , Lavorel S , Dray S , Reu B , Kleyer M , Wirth C , Prentice IC *et al*. 2016. The global spectrum of plant form and function. Nature 529: 167–171. 26700811 10.1038/nature16489

[nph70483-bib-0007] Falster D , Gallagher R , Wenk EH , Wright IJ , Indiarto D , Andrew SC , Baxter C , Lawson J , Allen S , Fuchs A *et al*. 2021. AusTraits, a curated plant trait database for the Australian flora. Scientific Data 8: 254. 34593819 10.1038/s41597-021-01006-6 PMC8484355

[nph70483-bib-0008] Kattge J , Bönisch G , Díaz S , Lavorel S , Prentice IC , Leadley P , Tautenhahn S , Werner GDA , Aakala T , Abedi M *et al*. 2020. TRY plant trait database–enhanced coverage and open access. Global Change Biology 26: 119–188. 31891233 10.1111/gcb.14904

[nph70483-bib-0009] Maitner B , Gallagher R , Svenning JC , Tietje M , Wenk EH , Eiserhardt WL . 2023. A Global assessment of the Raunkiæran shortfall in plants: geographic biases in our knowledge of plant traits. New Phytologist 240: 1345–1354. 37369249 10.1111/nph.18999

[nph70483-bib-0010] Poland TM , Patel‐Weynand T , Finch DM , Miniat CF , Hayes DC , Lopez VM . 2021. Invasive species in forests and rangelands of the United States: a comprehensive science synthesis for the United States forest sector. Berlin, Germany: Springer Nature, 455.

[nph70483-bib-0011] Pyšek P , Pergl J , Essl F , Lenzner B , Dawson W , Kreft H , Weigelt P , Winter M , Kartesz J , Nishino M *et al*. 2017. Naturalized alien flora of the world: species diversity, taxonomic and phylogenetic patterns, geographic distribution and global hotspots of plant invasion. Preslia 89: 203–274.

[nph70483-bib-0012] Seabloom EW , Borer ET , Buckley YM , Cleland EE , Davies KF , Firn J , Harpole WS , Hautier Y , Lind EM , MacDougall AS *et al*. 2015. Plant species' origin predicts. dominance and response to nutrient enrichment and herbivores in global grasslands. Nature Communications 6: 1–8. 10.1038/ncomms8710 PMC4518311 26173623

[nph70483-bib-0013] Siefert A , Violle C , Chalmandrier L , Albert CH , Taudiere A , Fajardo A , Aarssen LW , Baraloto C , Carlucci MB , Cianciaruso MV *et al*. 2015. A global meta‐analysis of the relative extent of intraspecific trait variation in plant communities. Ecology Letters 18: 1406–1419. 26415616 10.1111/ele.12508

[nph70483-bib-0014] Wang H , Harrison SP , Li M , Prentice IC , Qiao S , Wang R , Xu H , Mengoli G , Peng Y , Yang Z . 2022. The China plant trait database version 2. Scientific Data 9: 769. 36522346 10.1038/s41597-022-01884-4 PMC9755148

[nph70483-bib-0015] Westerband AC , Funk JL , Barton KE . 2021. Intraspecific trait variation in plants: a renewed focus on its role in ecological processes. Annals of Botany 127: 397–410. 33507251 10.1093/aob/mcab011 PMC7988520

[nph70483-bib-0016] Wright IJ , Reich PB , Westoby M , Ackerly DD , Baruch Z , Bongers F , Cavender‐Bares J , Chapin T , Cornelissen JHC , Diemer M *et al*. 2004. The worldwide leaf economics spectrum. Nature 428: 821–827. 15103368 10.1038/nature02403

